# Hyperspectral Attention Network for Object Tracking

**DOI:** 10.3390/s24196178

**Published:** 2024-09-24

**Authors:** Shuangjiang Yu, Jianjun Ni, Shuai Fu, Tao Qu

**Affiliations:** 1 Beijing Institute of Space Mechanics and Electricity, Beijing 100094, China; nijianjun@yeah.net (J.N.); fs0513@163.com (S.F.); 2 School of Computer Science, Wuhan University, Wuhan 430072, China; qutaowhu@whu.edu.cn

**Keywords:** hyperspectral videos, object tracking, multiscale features, spectral attention

## Abstract

Hyperspectral video provides rich spatial and spectral information, which is crucial for object tracking in complex scenarios. Despite extensive research, existing methods often face an inherent trade-off between rich spectral information and redundant noisy information. This dilemma arises from the efficient utilization of hyperspectral image data channels. To alleviate this problem, this paper introduces a hierarchical spectral attention network for hyperspectral object tracking. We employ a spectral band attention mechanism with adaptive soft threshold to examine the correlations across spectral bands, which integrates the information available in various spectral bands and eliminates redundant information. Moreover, we integrate spectral attention into a hierarchical tracking network to improve the integration of spectral and spatial information. The experimental results on entire public hyperspectral competition dataset WHISPER2020 show the superior performance of our proposed method compared with that of several related methods in visual effects and objective evaluation.

## 1. Introduction

Object tracking is a common task in diverse fields such as unmanned aerial vehicles [1], solar systems [2], vehicle traffic systems [3], and orientation estimation [4]. Given the initial bounding box, object tracking aims to continuously locate the target in videos [5,6,7,8]. Traditional algorithms like STC [9] and MOSSE [10] have achieved a balance between speed and performance in tracking, which has further propelled the development of single-object tracking tasks. These algorithms have also enabled the expansion of target tracking from visible [11] and infrared [12] modalities to hyperspectral modalities [13].

Hyperspectral imaging (HSI) has emerged as a transformative tool in object tracking. HSI offers unparalleled spectral resolution, enabling the discrimination of objects based on their unique spectral signatures [14,15]. Unlike traditional imaging with RGB images, HSI captures information across hundreds of spectral bands, which provides a detailed spectral profile for each pixel in the scene [16,17]. This rich spectral information is leveraged in various applications from environmental monitoring to military surveillance, identifying and tracking targets with high precision [18].

Existing hyperspectral object tracking methods exploit this spectral richness by employing techniques such as spectral angle mapping and anomaly detection to differentiate targets from their backgrounds [19]. Utilizing regions of interest provides benefits by focusing on the key information of the target [20,21,22], which helps to improve the accuracy of neural network coordinate regression to detect the target. However, these methods often struggle with high-dimensional data, spectral variability, and computational complexity, which limit their effectiveness in real-time applications [23]. Furthermore, most existing approaches fail to fully capitalize on the spatial information available in hyperspectral images [24]. Despite significant improvements, existing algorithms still face challenges in fully leveraging hyperspectral information and efficiently utilizing hierarchical multiscale features.

CNN-based methods have became a hot topic method in hyperspectral tracking [25]. Most CNN trackers are adapted from RGB trackers and focus primarily on spatial information, neglecting the rich spectral information in hyperspectral images [26]. HA-Net [27] is an anchor-free Siamese object tracking network that was specifically designed to process hyperspectral video data, but there are no specific experimental data or results. JSANet [28] jointly learns the Siamese network and the attention mechanism, allowing the two to work together and improve hyperspectral tracking performance. But, JSANet requires a large amount of annotated data to train the Siamese network. SMTN [29] uses multidimensional fusion and time-domain coding technology to improve the accuracy and robustness of target tracking in satellite videos. But, this method requires a large amount of annotated satellite video data to train the network. Sun [30] proposed a correlation-filter-based method for hyperspectral tracking that utilizes an image noise reduction technique based on principal component analysis with block-matching 3D (BM3D) for processing redundant information and further employs image fusion to improve target tracking in hyperspectral images. Zhao [31] integrated deep features with 3D edge features through convolution, which constructs an improved context filter to enhance object tracking accuracy in hyperspectral videos. SiamPKHT [32] focuses on transforming hyperspectral images into false-color images before extracting deep features to enhance object tracking performance.

RNN-based algorithms, particularly long short-term memory (LSTM) networks, have been explored to capture temporal dynamics in hyperspectral video tracking [33]. While they offer an advantages in tracking objects across sequences by utilizing temporal information, they similarly struggle with fully integrating spectral data into RNN-based frameworks. RNNs tend to focus on the temporal aspect at the expense of spatial and spectral resolutions. These approaches can lead to suboptimal tracking performance in environments where spectral distinctiveness is crucial for identifying the target [14].

However, two primary limitations emerge for existing deep-learning-based hyperspectral tracking algorithms: insufficient utilization of the full spectral range and ineffective integration of hierarchical multiscale features. These shortcomings result in compromised tracking accuracy, particularly in complex hyperspectral environments where the unique spectral signatures of targets are essential for reliable detection and tracking. There is a lot of redundant information and noise in hyperspectral images, making it necessary to also filter out the redundant noise.

**Figure 1:**
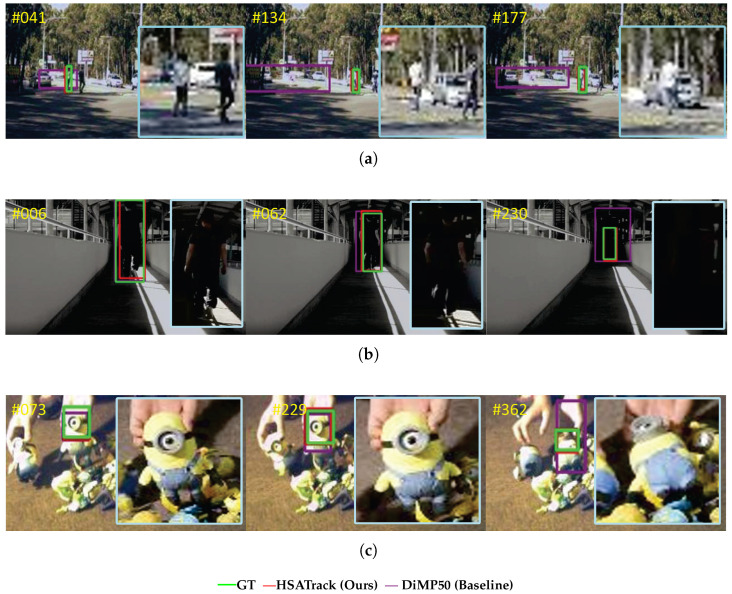
Visualization of results is presented to demonstrate the effectiveness of our method (**a**–**c**). Different colors represent different algorithm tracking results. (**a**) The tracking comparison in “White Man” video sequence, (**b**) the tracking comparison in “Man In Hallway” video sequence, and (**c**) the tracking comparison in “Toy” video sequence.

To address the critical challenges of the underutilization of multispectral data and the redundant information in hyperspectral images, we propose a hierarchical structure based on spectral attention for hyperspectral object tracking, named HSATrack. HSATrack incorporates a multilevel spectral channel attention mechanism with an adaptive soft threshold. This attention mechanism with a soft threshold in HSATrack not only enhances the robustness of feature extraction across diverse spectral bands but also filters out redundant noise. By meticulously integrating spectral data, HSATrack provides a solution to a previously unaddressed gap in multispectral target tracking. A visual comparison of our method, ground truth, and our baseline DiMP50 [34] is presented in Figure 1, illustrating the comparative visualization of our HSATrack method against the ground truth (GT) and the baseline DiMP50. HSATrack performs tracking processing based on hyperspectral images, which contain rich spectral information. The effectiveness of HSATrack is particularly evident in frames 041 and 134, where the tracker accurately delineates the target in urban environments with complex and dynamic backgrounds. Our proposed HSATrack contributes the following:

- In order to make full use of spectral information and explore the correlation between different spectral bands, we introduced a multispectral band attention mechanism with an adaptive soft threshold, called SAM. SAM is integrated into the backbone to improve the ability of model to extract information from spectral segments and eliminate redundant information in multispectral bands.
- In order to make full use of hyperspectral information at different spatial scales, we embeded the multispectral attention mechanism into the backbone network layer by layer to construct a hierarchical feature extraction backbone network.
- The experiments on the only available hyperspectral competition dataset WHISPER2020 demonstrated promising results for our HSATrack.

## 2. Related Work

Hyperspectral imaging, capturing information across a wide range of the electromagnetic spectrum, has gained significant attention in fields like remote sensing, agriculture, and environmental monitoring. Recently, integrating hyperspectral data with video sequences has led to the emergence of hyperspectral video analysis. A key challenge in this analysis is tracking targets within hyperspectral video frames. In Section 2.1, we introduce some related work regarding traditional algorithms, and we further introduce some CNN trackers within the deep learning algorithms in Section 2.2.

### 2.1. Traditional Algorithms

Traditional algorithms have been widely explored for processing hyperspectral video images. Principal component analysis (PCA) is a commonly used method for reducing dimensionality and has been applied to hyperspectral data [35]. This transformation effectively reduces computational complexity while preserving essential spectral information. As a result, it enables real-time object tracking in hyperspectral videos.

Researchers have applied spectral unmixing techniques to hyperspectral video object tracking and detection. Spectral unmixing is a mathematical algorithm that distinguishes multiple fluorophore signatures within a multicolor tube and places each signature into its own parameter [36]. These methods aim to break down mixed spectral signatures into their component materials, which makes it easier to identify objects with unique spectral properties. Non-negative matrix factorization and linear spectral unmixing are popular techniques used for hyperspectral video analysis. These techniques allow for accurate object detection in complex scenes. MHT [37] integrates the features derived from the fractional presence of materials and the spectral–spatial distribution of gradients across multiple dimensions. Subsequently, these features are utilized by a tracker that is sensitive to the background context. Despite the remarkable performance exhibited by these algorithms, their dependency on manually crafted features constrains their capacity to thoroughly identify and define objectives within the scope of hyperspectral video footage. Qian [38] built a set of 3D cubic group filters using the principle of correlation filtering to boost the performance of hyperspectral image target tracking algorithms. ASSDA [39] constructs a complex background recognition module based on correlation filters, which enhances the tracking performance of hyperspectral target trackers under interference from cluttered backgrounds.

### 2.2. Deep Learning Algorithms

DeepHKCF [40] converts hyperspectral videos into pseudo-color images by synthesizing three bands like RGB imagery. This method adopts the existing AlexNet model to extract features, which are then input into the KCF tracker. Li et al. [41] introduced a novel 3D CNN architecture for detecting objects in hyperspectral videos. This architecture utilizes both spatial and spectral information and shows high accuracy in identifying objects under various lighting conditions. BAE-Net [42] features a band selection module that learns different band combinations, each creating a pseudo-color image. This approach produces multiple pseudo-color images from the same hyperspectral video frame and processes them with the VITAL [43] tracker. This results in a combined final tracking result for the frame. Building on BAE-Net, SST-Net [44] adds spatial and temporal attention mechanisms to enhance the band selection module’s efficiency. SiamHYPER [13] introduces a template-enhanced hyperspectral target tracker using a Siamese architecture. However, deep-learning-based hyperspectral object tracking, mainly through adapting pseudo-color video synthesis from three hyperspectral bands using existing RGB-based trackers, faces challenges. The “data hungry” nature of these approaches means they struggle when developing a generalized feature representation or achieving high predictive accuracy with limited training samples. This limitation affects the use of high-level hyperspectral semantic features and the accuracy of bounding boxes in hyperspectral tracking. Trans-HST [45] mitigates information loss from hyperspectral image data by constructing a feature fusion module based on Transformer [46]. TBR-Net [45] leverages the long-range capabilities of Transformer for the context modeling of target information to enhance the performance of hyperspectral target tracking. In older to reduce the computational complexity of the Transformer structure in hyperspectral target tracking algorithms, MambaHsi [47] introduces the Mamba [48] architecture to achieve linear computational complexity while maintaining highly competitive tracking performance.

Zhang et al. [49] introduce a notable approach by integrating recurrent neural networks (RNNs) with CNNs, capturing temporal dependencies in hyperspectral video frames. Their model focuses on the temporal evolution of spectral signatures, achieved robust object detection in dynamic scenes. Additionally, incorporating attention mechanisms in hyperspectral object detection [50] enhances the model’s ability to focus on relevant spectral bands. This results in improved accuracy and efficiency.

## 3. Methodology

**Figure 2:**
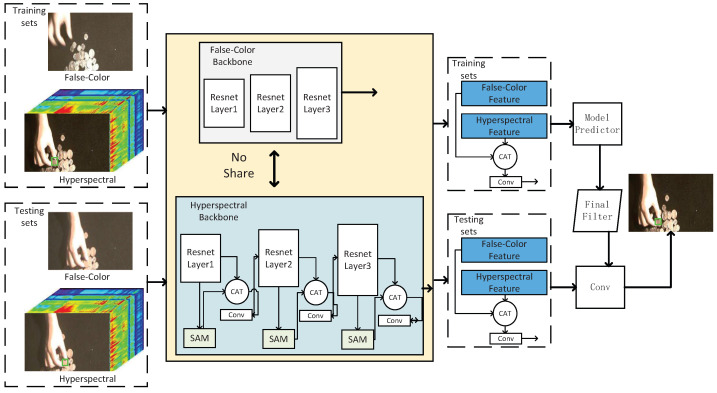
Illustration of our HSATrack, which includes three main components: the false-color-based backbone, hyperspectral backbone, and SAM. In the training and test sets, pairs of false-color-based images and hyperspectral images are input into separate backbone networks for feature extraction. False-color images and hyperspectral images do not share the same backbone network. During feature extraction, they are processed through their respective extraction backbone networks.

The architecture of our HSATrack is illustrated in Figure 2. This section provides an overview of both DiMP and HSATrack. HSATrack builds upon the foundation of DiMP50 [34], as detailed in Section 3.1, where we describe the architecture of our method and DiMP50 comprehensively. In Section 3.2, we discuss the hierarchical backbone of our method in detail. Section 3.3 outlines the construction of the spectral attention module (SAM), which features an attention mechanism module tailored for spectral channels. This module dynamically adjusts the weights of different spectral channels using an attention mechanism, emphasizing crucial spectral information for hyperspectral tracking tasks. Additionally, an adaptive soft threshold in SAM helps filter redundant information across multispectral bands. In Section 3.4, we integrate the multispectral channel attention module into the RGB-adapted backbone network. We progressively add the multispectral attention module layer by layer within the backbone network, creating a hierarchical multispectral attention feature extraction network. This approach maximizes the complementary information from different spectral bands in the hyperspectral image. Lastly, Section 3.5 elaborates the multistep training strategy of our HSATrack.

### 3.1. Algorithm Overview

Our method uses a hybrid approach that combines discriminative and generative modeling techniques, which is based on DiMP. DiMP dynamically updates the target model through online learning. The algorithm adapts to changes in target appearance and combines multiresolution features for more accurate dynamic localization. It uses information from different channels for precise predictions and performs well across various datasets. In this section, we delve into the benefits of adaptive discriminant feature learning, online updating of the generative model, and this approach. We also present the framework of this method.

For adaptive feature learning, we use deep neural networks to identify distinctive features of the target object. These features highlight crucial visual signals and allow the algorithm to differentiate the target from its background effectively. Training the model on extensive datasets guarantees its generalization capability across different object types and environments.

Our method’s generative model adapts to the target’s motion and appearance changes over time for online training and updates. HSATrack consistently estimates the target’s position by modeling these uncertainties probabilistically, proving reliable even in difficult situations. This adaptability renders our model exceptionally effective at tracking objects with intricate motion behavior.

HSATrack advances hyperspectral tracking by cleverly combining an adaptive channel shrinkage network with the proven DiMP framework. This integration enhances signal quality by eliminating noise and capitalizing on vital spectral information, facilitated by its adaptive soft thresholding. HSATrack retains DiMP’s real-time response capacity and durability, making it suited for tracking in dynamic and complex settings, such as robotics and security.

### 3.2. Backbone from RGB

We initialize our hyperspectral input backbone using a pretrained backbone model. The DiMP50 model [34], as provided by the corresponding author, serves as the standard model and did not require retraining. During feature extraction on the backbone network, both the training sets and the test sets contained false-color images and hyperspectral images. The false-color images and hyperspectral images from both sets input the false-color backbone network and the hyperspectral backbone network for feature extraction, respectively. Finally, the extracted features of the false-color and hyperspectral images from the training and test sets are then fused through channel concatenation, which serves as the input of the model predictor and convolution calculation.

As shown in Figure 2, we select three bands from the hyperspectral imagery to create a three-band image that serves as the input for the RGB-based DiMP50 [34] backbone. Next, we replicate and expand the number of channels in the input layer of the backbone network of the three-channel false-color-based DiMP50 [34] network model from 3 to 16. This adjustment accommodates the number of input channels required for hyperspectral imagery, thereby establishing the hyperspectral feature extraction backbone network.

### 3.3. Spectral Attention Module

**Figure 3:**
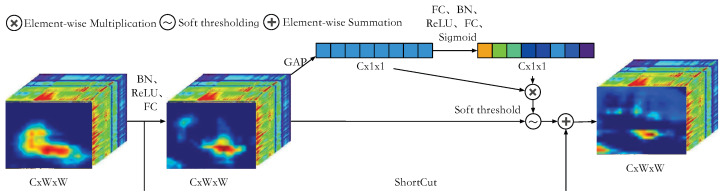
Illustration of SAM in our HSATrack. Our SAM mainly performs attention weighting processing on hyperspectral image features. In order to suppress noise, we introduce adaptive soft threshold processing operations. Adaptive soft threshold processing uses the learning ability of deep network channel attention to learn an adaptive threshold.

When processing hyperspectral images, the unique characteristics of spectral channels convey rich information at various wavelengths. To effectively harness this wealth of information, we introduce a hyperspectral channel attention mechanism. This mechanism can adaptively learn the weight of each channel, emphasizing the crucial spectral features within the model. Figure 3 illustrates the architecture of the spectral attention module (SAM). Our multispectral channel attention module operates in three stages: feature integration in Section 3.3.1, calculation of spectral channel attention weights in Section 3.3.2, and the application of attention weights in Section 3.3.3. These stages are described below.

#### 3.3.1. Feature Integration

We start by extracting features from the hyperspectral data through several convolutional layers. Assume that the input hyperspectral feature is represented by $Hyper\in R^{N\times C\times W\times H}$, where *N* is the batch size, *C* is the number of hyperspectral feature channels, H and W are the height and width of the hyperspectral feature, respectively. We use a convolutional layer to map the hyperspectral features to a new integrated feature space $Z\in R^{N\times D\times W\times H}$, where *D* is the number of integrated feature channels.

#### 3.3.2. Spectral Channel Attention Calculation

In this step, we introduce the spectral channel attention mechanism whose purpose is to learn the weight of each spectral channel to highlight important spectral information. The operating formula is presented as follows:

(1) Spectral_Attention_Weights=Softmax(FFN(GAP(Z))),

where *Z* is the integrated feature tensor in Section 3.3.1, GAP is the global average pooling (GAP) operation to reduce the dimensionality of *Z*, and FFN is the feed-forward neural network (FFN) to calculate the attention weight for each spectral channel. The Softmax function acts as an activation function, ensuring that the attention weights sum to 1, thus forming a valid probability distribution.

#### 3.3.3. Attention Weight Application

Finally, we use the calculated weight of attention to weight in this step and sum the original feature tensor *Z* according to the spectral channel to obtain the weighted feature tensor:

(2) $$X_{weighted}=\sum_{i=1}^{C}Spectral\_Attention\_Weights_{i}\times Z_{i},$$

where $Spectral\_Attention\_Weights_{i}$ is the *i*-th element of the attention weight, $X_{weighted}$ is the weighted feature tensor, and $Z_{i}$ is the *i*-th channel of the feature tensor *Z*.

In SAM, the attention mechanism enables the network to automatically learn and focus on the most important spectral channels in the input hyperspectral image. This mechanism better captures key information in the data.

Hyperspectral imagery is rich in data pertaining to targets but is also characterized by significant redundancy. To improve the clarity of target depiction and maximize the use of distinctive data, it is crucial to minimize the spread of superfluous data while maintaining the clarity of the target’s features. To this end, we implement a soft thresholding technique that converts noisy data into less significant areas with values near zero. Subsequently, we eliminate features with minimal values and scale down features with values further from zero to meet the threshold criteria [51,52]. The function of the soft threshold is as follows:

(3) $$y=\left\{\begin{array}{cc} x-\tau , & x>\tau ,\\ 0, & |x|\leq \tau ,\\ x+\tau , & x<-\tau , \end{array}\right.$$

where *x* is the input tensor, *y* is the output tensor, and τ is the soft thresholding threshold. Determining a suitable threshold for the soft thresholding process is a complex task, as the ideal threshold can differ significantly across various image types and data modalities. Th edeep learning architecture facilitates the automatic learning of parameters, inspiring us to incorporate soft threshold mechanisms into deep learning tracking networks. Compared to other attention algorithms used in target tracking [53,54], our proposed soft threshold channel attention can amalgamate essential channel weight information. It also utilizes adaptive soft thresholds to eliminate unnecessary redundant channel information, thus efficiently processing hyperspectral image features. The specific operating formula is presented as follows:

(4) $$\begin{array}{cc} \tau & =Sigmoid(X_{weighted}), \end{array}$$

(5) $$\begin{array}{cc} SAM_{out} & =X_{weighted}+X_{weighted}⊗\tau , \end{array}$$

where $X_{weighted}$ is the weighted feature tensor in Section 3.3.3, Sigmoid acts as an activation function, and $SAM_{out}$ represents the output of the SAM module.

Traditional channel attention mechanisms typically use the sigmoid or ReLU functions, which lack flexibility as they uniformly process all input channels. In contrast, the adaptive soft thresholding function can dynamically adjust the threshold τ, thereby more effectively distinguishing important features from noise.

Specifically, for a given input z, the output of CA (channel attention) is:

(6) $$CA_{out}=\sigma \left(Wz+b\right),$$

where σ is a sigmoid or ReLU function, W represents the model weight, and b is the biases.

For SAM, the output is

(7) $$SAM_{out}=f\left(Wz+b,\tau \right)$$

Because f(x,τ) is more flexible, it can dynamically adjust τ to better select important features. This manifests as the stronger suppression of small feature values and the retention of more information when feature values are large. In the presence of noise, traditional channel attention mechanisms may fail to effectively suppress noise, resulting in reduced feature extraction performance. The adaptive soft thresholding, by dynamically adjusting the threshold, can more effectively suppress noise.

Assuming the presence of noise n in the input features, the output of CA is

(8) $$CA_{out}=\sigma \left(W\left(z+n\right)+b\right)$$

where σ is sa igmoid or ReLU function, W represents the model weight, and b is the biases.

For SAM, the output is

(9) $$SAM_{out}=f\left(W\left(z+n\right)+b,\tau \right)$$

Since the value of n is typically small, the adaptive soft thresholding function strongly suppresses it, thereby reducing the impact of noise. In summary, through mathematical formulations, we can prove that SAM has significant advantages over the traditional channel attention in terms of feature selection and noise suppression. These advantages are mainly due to the dynamic adjustment capability of the adaptive soft thresholding function, which can adaptively select thresholds based on different input features, thereby more effectively extracting important features and suppressing noise.

### 3.4. Hierarchical Spectral Attention Feature Extraction Module

In this section, we design an asymmetric structure for the backbone network’s feature extraction. The backbone network for false-color-based images retains the same structure as Resnet50 [55], which serves as our baseline network, along with DiMP50 [34]. For the 16-band hyperspectral image, we create a backbone network according to the design in Section 3.2. We then add our multispectral attention mechanism layer by layer to the backbone network. After concatenating the features extracted by the layer-by-layer attention mechanism are concatenated, they are returned to the original channel through a convolutional structure. This process realizes hierarchical multispectral information integration and utilization.

As depicted in Figure 3, the convolution processing operation of the *l*-th layer of the backbone network can be expressed as

(10) $$Out^{l}=Cat\left(SAM\left(Resnet\_Layer\left(Out^{l-1};\theta_{l}\right)\right),Out^{l-1}\right),$$

where $Out^{l}$ is the out feature tensor of the *l*-th layer, $Out^{l-1}$ is the out feature tensor of the l−1-th layer, $\theta_{l}$ represents the convolution parameter of the *l*-th layer, SAM denotes the SAM in Section 3.3.2, and Cat is the concatenate operation.

### 3.5. Multiatep Training Strategy

The main challenge in multispectral image analysis lies in the scarcity of pretrained network models specifically designed for multispectral data. This issues arises from the limited availability of large-scale annotated multispectral datasets, which are essential for training deep learning models. Consequently, initializing a feature extraction network for multispectral images from scratch often leads to suboptimal performance due to the insufficient generalization capability of the network, attributed to the lack of diverse and comprehensive feature representations learned during training.

**Figure 4:**
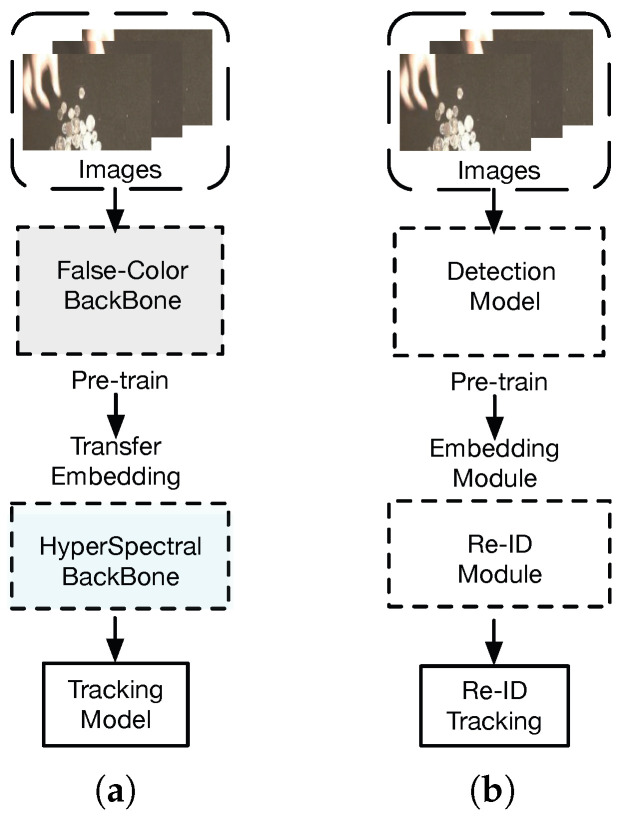
Framework comparison of the proposed method HSATrack with the re-OBJ training framework, which uses the same multistep training strategy. (**a**) The multistep training process of the proposed method. (**b**) The training process of re-OBJ, which also uses a multistep training strategy. (**a**) “Training for HSATrack”. (**b**) “Training for re-OBJ”.

**Figure 5:**
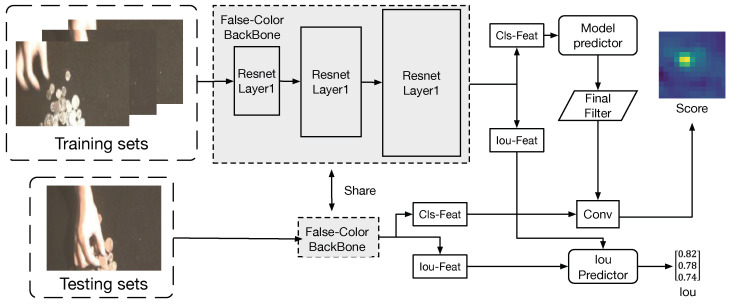
Illustration of the first step in our HSATrack’s multistep training strategy. At this stage, we train a complete DiMP50 network model using false-color-based images.

**Figure 6:**
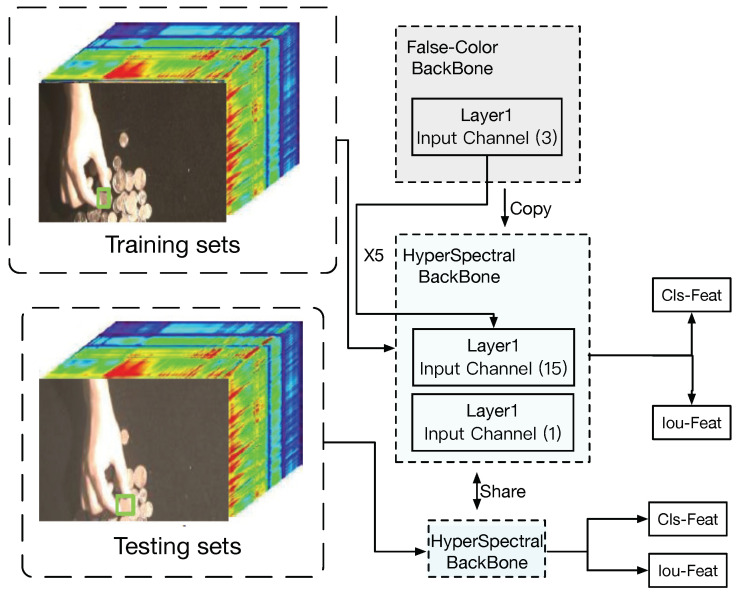
Illustration of the second step of our HSATrack in the multistep training strategy. At this stage, we use the trained false-colour-based backbone model to initialize the hyperspectral backbone model and copy the input layer parameters from 3 layers to 16 layers. After training the two backbone networks, freeze the parameter weights of all networks.

The absence of dedicated pretrained models presents a significant challenge in multispectral image feature extraction. To address this issue, utilizing pretrained models from RGB image datasets proves beneficial. Models trained on comprehensive datasets like ImageNet have acquired the capability to recognize a broad array of features. Transferring this knowledge to multispectral image networks offers a head start, aiding them to achieve faster convergence and potentially enhancing performance, especially in scenarios where multispectral training data are limited. This strategy leverages the generalization abilities garnered from RGB data, laying a solid foundation for feature extraction from multispectral images. To acquire a multispectral backbone network model apt for 16 bands, we adopt a multistep training approach. As is shown in Figure 4, drawing inspiration from methods such as Fast re-OBJ [56] and IO-ReID [57], we also apply the technique of loading pretrained network models for training the hyperspectral backbone feature extraction network in this article. However, differing from Fast re-OBJ and IO-ReID, which view the tracking task as a blend of target detection and re-identification tasks by integrating the pretrained target detection network with the re-identification task, our approach initially trains a three-channel false-color backbone. We then incorporate the pretrained false-color backbone model to initialize the hyperspectral backbone feature extraction network, performing further training to accomplish the target tracking task. This method enhances the training efficiency of the tracking task and diverges from the conventional detection and re-identification framework. The training flow chart is displayed in Figure 5. Our strategy is bifurcated into two phases. The first step is training the backbone network $Backbone_{false-color}$ on false-color-based images; as is shown in Figure 6, the second step is training $Backbone_{hyper}$, extended in Section 3.2, using 16 spectral band images for training. The above two steps are consistent with DiMP50’s [34] training, except for the learning rate. We use the backbone trained on RGB dataset as a pretrained model and then initialize the hyperspectral feature extraction backbone network by copying the model parameters to meet the number of channels of the hyperspectral input image. In the same way, when other modalities need to be processed, such as thermal infrared images, the RGB feature extraction backbone can be used as a pretraining model to process infrared modalities in the same way.

## 4. Experiments and Results

We conducted extensive experiments on the hyperspectral WHISPER2020 dataset to evaluate our tracker. We introduce the implementation details and the tracking process of our HSATrack in Section 4.1. In Section 4.2, we illustrate the evaluation data and metrics. Section 4.3 offers a numerical evaluation of our tracker’s performance in comparison with that of existing hyperspectral tracking solutions, complemented by a qualitative assessment. Section 4.4 discusses instances where HSATrack encounters tracking difficulties. To conclude, we perform ablation analysis by examining various versions of HSATrack, thereby validating the efficacy of our approach.

### 4.1. Implementation Details and Tracking Process

Our network was implemented by Python 3.7 and PyTorch 1.8.2 on a machine with an Intel i9-12900 K processor (Intel, Santa Clara, CA, USA), 64 GB RAM, and an NVIDIA GeForce RTX 3090 GPU (NVIDIA, Santa Clara, CA, USA). We used ADAM [58] with a learning rate of 1 × 10^−4^ and a learning rate decay of 0.2 every 15th epoch. The image patches in the training sets and testing sets were extracted by sampling a random translation and scale relative to the target annotation. We set the base scale to 5 times the target size to incorporate significant background information. For each sequence training, we sampled 3 test and train frames, using a segment length of 60 frames. Additionally, we employed a novel image preprocessing scheme and workflow to boost tracking performance. This preprocessing not only normalized the input data but also enhanced the model’s ability to differentiate the target object from its surroundings, essential for accurate tracking across various scenarios. Preprocessing started with cropping the image region around the anticipated target location, concentrating the algorithm’s focus near the object and minimizing the computational effort by disregarding irrelevant image sections. We then resized the cropped image patches to uniform dimensions, a crucial step to ensure consistent input scale for the tracker, aiding in object tracking across frames despite size changes. To further increase the model’s resilience to changes in object appearance and environmental conditions, we applied image augmentation techniques such as rotation, scaling, blurring, and brightness adjustment. This approach created a varied set of training samples from the existing data, mimicking various tracking conditions.

The parameter amount of our network model was 2.3 G, our network occupied 14 GB of GPU memory during training, and the training time of the network was 18 h. And, we tested our proposed HSATrack on the WHISPER2020 training set. HSATrack is compatible with DiMP [34] testing sets and achieved a performance of 41 fps.

### 4.2. Dataset

In this section, we detail the dataset and the significance of the evaluation metrics, including composition, attributes, and annotation in Section 4.2.1, and metrics in Section 4.2.2.

#### 4.2.1. The Composition, Attributes, and Annotation of the Dataset

The WHISPER2020 dataset features a wide range of video sequences, each posing distinct challenges for object tracking. These sequences include scenarios with camouflaged targets or complex background clutter, featuring objects such as cards, coins, soda cans, fruit, paper, pedestrians, students, and various toys. The dataset is organized into three specific types of videos. The first is (i) Hyperspectral Video Data. This category includes video sequences captured with hyperspectral imaging technology, offering detailed spectral information for each pixel; (ii) False-Color Video Data. Derived from hyperspectral sequences, these videos present the same scenes in a false-color format, enabling advanced analysis based on particular spectral bands; (iii) RGB Video Data. Recorded concurrently with the hyperspectral sequences, these videos provide the standard RGB color information, captured at the same time and from nearly identical viewpoints as the hyperspectral videos.

The WHISPER2020 hyperspectral dataset requires several preprocessing steps to ensure raw data quality and usability. These steps include data reading from native formats, radiometric correction to convert raw data into physical reflectance values, and geometric correction to align images with geographic coordinates. Noise removal techniques, such as dark current correction, stripe noise correction, and stray light correction, are applied to enhance the signal-to-noise ratio. Spectral calibration adjusts for wavelength shifts, and data normalization mitigates illumination variability. Spatial filtering smooths the images, and dimensionality reduction techniques like PCA reduce data complexity. Finally, spectral feature extraction prepares the dataset for analysis, which is followed by the creation of labeled training and testing sets for machine learning applications.

The dataset includes a total of 40 video sets for training and 35 sets for testing. Each video is labeled with specific challenging factors chosen from a set of 11 attributes. Moreover, each frame within the dataset was precisely annotated with bounding boxes to outline the targets by Xiong et al. [37]. Notably, the labels for hyperspectral and RGB videos were generated independently to ensure the accuracy of annotations for both types of data. Additionally, the labels for hyperspectral videos are directly applicable to false-color videos, which maintain consistency across different representations of the same scenes.

#### 4.2.2. Evaluation Metrics

In the evaluation of object trackers, several key metrics were employed, including precision plots, success plots, distance precision (DP) scores, and area under the curve (AUC) scores, which are crucial for assessing the performance of tracking algorithms. The precision plot (PP) can be described as $PP=\frac{1}{N}\sum_{i=1}^{N}1\left(d_{i}<\tau \right)$, where *N* is the total number of frames, $d_{i}$ is the distance between the predicted and ground-truth object centers in the *i*th frame, and τ is a predefined threshold, which is 20 in the WHISPER2020 dataset. Precision plots are utilized to demonstrate the proportion of frames in which the estimated positions of the objects are within a predefined distance threshold from the actual ground-truth centers. Specifically, the DP rate, which represents the average precision at a 20-pixel threshold, is a commonly reported measure. On the other hand, success plots illustrate the percentage of frames where the overlap ratio between the predicted and the actual bounding boxes surpasses a certain threshold, which varies from 0 to 1. The success plot is given by $SP=\frac{1}{N}\sum_{i=1}^{N}1\left(o_{i}>\theta \right)$, where *N* is the total number of frames; $o_{i}$ represents the overlap ratio between the predicted and ground-truth bounding boxes in the *i*-th frames. The AUC score, derived from the success plots, quantifies the overall performance over all thresholds.

### 4.3. Comparison with Other Methods

**Figure 7:**
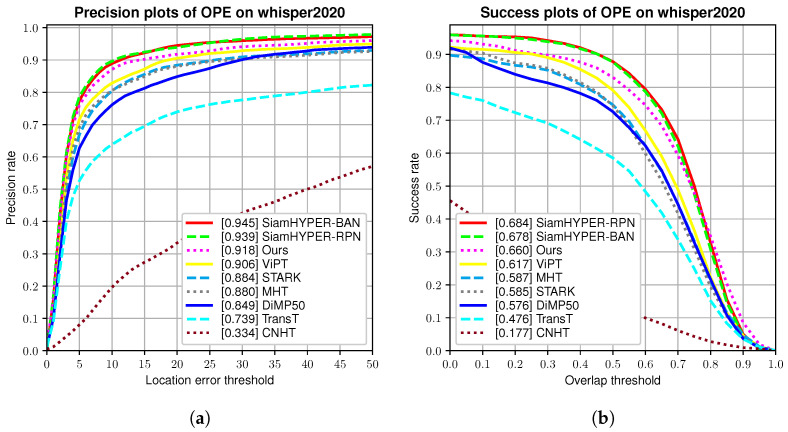
Precision and success rate plots for hyperspectral trackers on WHISPER2020. (**a**) The AUC curves on WHISPER2020, and (**b**) the DP curves on WHISPER2020. The *y*-axis indicates the proportion of frames meeting various thresholds in the dataset. The *x*-axis in (**a**) shows different center point distance thresholds, while the *x*-axis in (**b**) represents different overlap thresholds.

For a fair comparison, we compare our HSATrack with other hyperspectral trackers in Figure 7. This includes HSATrack, our baseline DiMP50 [34], TransT [11], IRSTNet-based tracker [12], STARK [59], ViPT [60], SiamHYPER_BAN [13] (SiamHYPER is a tracker based on SiamBAN [61]), SiamHYPER_RPN (SiamHYPER is a tracker based on SiamRPN++ [8]), BAE-Net [42], MHT [37], and CNHT [38], tested on hyperspectral videos. For testing general RGB target tracking algorithms such as TransT, we only used the pseudo-color images from the WHISPER2020 dataset for inference testing. We also provide a comparison of tracking performance and speed in Table 1.

#### 4.3.1. Quantative Evaluation

As is shown in Figure 7, the proposed HSATrack method obtains the third-best AUC score of 0.660 and the third-best DP of 0.918. The performance of our method in terms of AUC and DP is far inferior to that of SiamHYPER_BAN [13] and SiamHYPER_RPN [13], but, as shown in Table 1, our method is faster, and the speed of our method far exceeds that of SiamHYPER_RPN [13]. In Table 1, the IRSTNet-based tracker shows the fastest speed thanks to the lightweight design of the algorithm, HSATrack far exceeds our baseline DiMP50 [34] method in performance, and our proposed method exceeds DiMP50 [34] by 0.084 on the AUC and 0.069 on the DP, which achieves a joint second-ranked speed on the hyperspectral videos. HSATrack surpasses TransT [11], STARK [59], and ViPT [60] in the AUC and DP scores. These transformer-based tracking algorithms require a large amount of data for training and fitting, and the hyperspectral training set is small. The method we propose does not rely on many datasets, so the performance of our method far exceeds those of these algorithms. The module for real-time spectral data refreshes the object’s profile while it moves, ensuring it stays aligned with any alterations in shape and spectral properties. In the SiamHYPEK [13] framework, this module has been upgraded to the SSATTN module, which is designed to be more efficient, capable of boosting processing speed by five frames per second with a minimal impact on the AUC score, decreasing it by just 0.9%. The SiamHYPER_BAN [13] technique surpasses the leading BAE-Net [36] tracker in the field of hyperspectral tracking, achieving a 7.2% and 6.8% improvement in AUC and DP metrics, respectively. However, these methods are not as fast as our proposed method. Due to the addition of more modules and the non-hared dual-backbone feature extraction network, our speed drops slightly compared to our baseline DiMP50 but still qualifies as real-time processing.

We present a comparison of frames per second (FPS) in Table 1. DiMP50_*our*_ refers to the results achieved by training on the WHISPER2020 dataset using the original hyperparameters of the DiMP50 tracker [34]. DiMP50_*our*_ achieves a processing speed of 46 FPS on the GPU, making it the fastest hyperspectral tracker. HSATrack operates at 34 FPS on the GPU, ranking it as the second-fastest hyperspectral tracker. BAE-NET, an enhancement in the RGB-based VITAL tracker [43], is capable of running at 1.5 FPS on the GPU. Incorporating a band selection module and using multiple pseudo-color images for prediction limit BAE-Net’s GPU processing speed to 0.5 FPS. MHT; using SSHMG features and hyperspectral unmixing abundances to characterize hyperspectral targets achieves a CPU processing speed of 0.5 FPS. CNHT, which employs fixed convolution kernels for feature extraction from hyperspectral imagery and inputs them into the KCF tracker, reaching a CPU processing speed of 0.8 FPS. The proposed method significantly surpasses the existing hyperspectral trackers in terms of processing speed. Except for HA-Net, the current hyperspectral trackers do not exploit high-level semantic features.

We further provide an AUC comparison of properties in Table 2, including Background Clutter (BC), Deformation (DEF), Fast Motion (FM), Illumination Variation (IV), In-Plane Rotation (IPR), Low Resolution (LR), Motion Blur (MB), Occlusion (OCC), Out-of-Plane Rotation (OPR), Out-of-View (OV), Scale Change (SC), and Scale Variation (SV). Among the results for the hyperspectral tracking in terms of AUC, the proposed method HSATrack obtains promising results. HSATrack obtains an AUC score of 0.653 on SV, which surpasses the results of the baseline RGB-based tracker (DiMP50 [34]) by 0.113%, but is inferior to SiamHYPER_BAN [13], which is 0.075 points worse than SiamHYPER_BAN [13]. HSATrack obtains an AUC score of 0.66 on SC but is inferior to SiamHYPER_BAN [13], which is 0.107 worse than SiamHYPER_BAN [13]. HSATrack obtains an AUC score of 0.585 on OV but is inferior to SiamHYPER_BAN [13], which is 0.11 worse than SiamHYPER_BAN [13]. HSATrack obtains an AUC score of 0.719 on OPR but is inferior to SiamHYPER_BAN [13], which is 0.033 worse than SiamHYPER_BAN [13]. HSATrack obtains an AUC score of 0.651 on IV, which surpasses the results of the baseline RGB-based tracker (DiMP50 [34]) by 0.105%, and surpasses the SiamHYPER_BAN [13] by 0.094%. HSATrack obtains an AUC score of 0.778 on DEF, which surpasses the results of the baseline RGB-based tracker (DiMP50 [34]) by 0.092%, and surpasses the SiamHYPER_BAN [13] by 0.008%. HSATrack obtains an AUC score of 0.685 on BC, which surpasses the results of the baseline RGB-based tracker (DiMP50 [34]) by 0.084% but is inferior to SiamHYPER_BAN [13], which is 0.017% worse than SiamHYPER_BAN [13].

**Figure 8:**
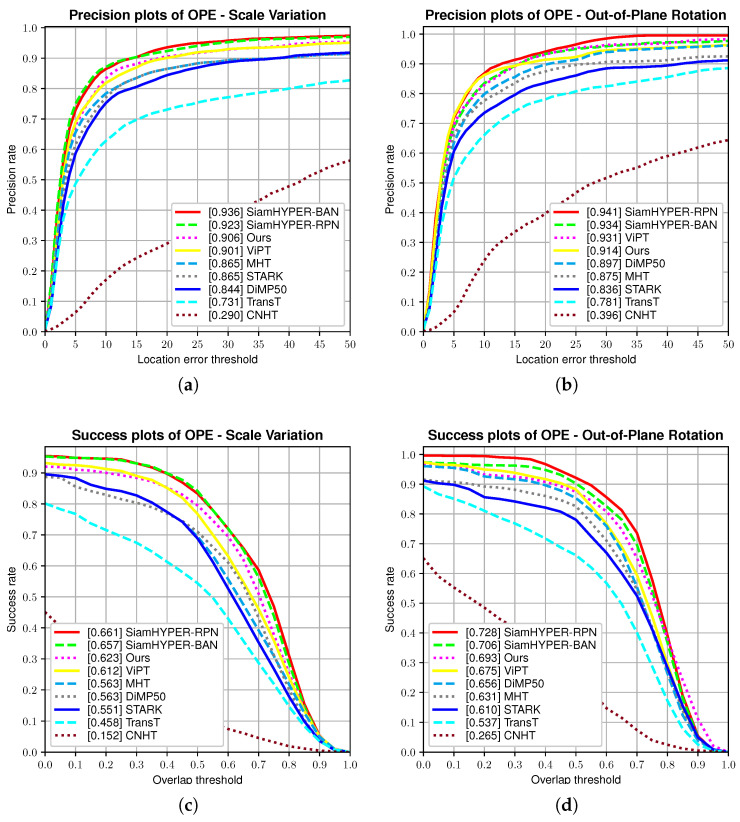
Attribute comparison of the hyperspectral trackers. (**a**,**b**) The AUC curves on the SV and OPR attributes. (**c**,**d**) The DP curves on the SV and OPR attributes.

We provide a detailed AUC and DP curve comparison in Figure 8, including Scale Variation (SV) and Out-of-Plane Rotation (OPR). In the SV curve, our method ranks second, below SiamHYPER_RPN [13], significantly surpassing our baseline DiMP50. At low overlap rates, our method leads but performance significantly drops with increasing overlap ratio, indicating a need for further optimization in the regression component. This trend is consistent across the other attribute curves in AUC, setting our next focus on optimizing overlap rates. In the OPR attribute of the DP curve, our method and the others perform well, suggesting the DP attribute is not overly challenging in this dataset. This observation highlights the WHISPER2020 dataset’s limitations, pointing to the need for larger and more comprehensive datasets for further exploration.

#### 4.3.2. Qualitive

**Figure 9:**
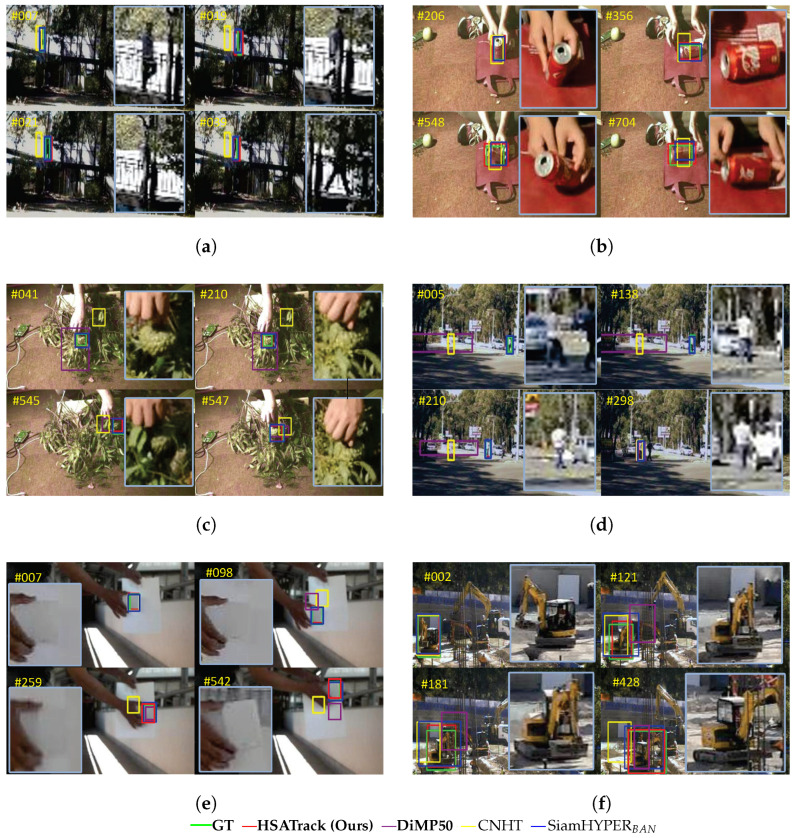
Qualitative evaluation of HSATrack, ground truth, and three state-of-the-art trackers: our baseline DiMP50, CNHT, and SiamHYPER_*BAN*_. The target in sequence (**a**) faces occlusion and scale variation challenges. The targets in sequences (**b**,**c**,**e**) primarily face challenges with scale variation and out-of-plane rotation. And, the target in sequence (**d**,**f**) mainly involves a scale variation challenge. (**a**) “Campus” video sequence. (**b**) “Can” video sequence. (**c**) “Toy” video sequence. (**d**) “White Man” video sequence. (**e**) “Card” video sequence. (**f**) “Excavator” video sequence.

In Figure 9, we present the qualitative results of our method HSATrack, the ground truth, our baseline DiMP50 [34], CNHT [38], and SiamHYPER_*BAN*_ [13]. The results demonstrate HSATrack’s superior capability to manage scenarios with rapid movement, motion blur, camera shake, and object deformation. Specifically, in the video segment highlighted in Figure 9, the target experiences significant distortion due to motion blur and deformation, leading to tracking errors for the other algorithms. In contrast, HSATrack successfully mitigates the impact of such distortions, maintaining a precise and tight tracking box around the target. Similarly, in instances where the camera moves, HSATrack continues to accurately track the target, outperforming the other methods that struggle in these conditions.

The adaptive soft thresholding channel attention mechanism introduced in HSATrack has shown improvement in instances where hyperspectral object tracking traditionally struggles. As shown in Figure 9a,f, in scenes with variable lighting conditions or camouflaged objects, the mechanism has been particularly effective in enhancing feature discernment. The attention model adaptively accentuates informative spectral bands while suppressing less relevant ones, thereby improving the signal-to-noise ratio for the tracker.

In practical applications, the impacts of HSATrack are manifold. As shown in Figure 9e, in precision agriculture, it could enable more accurate tracking of plant health by focusing on spectral bands that indicate disease or stress, even in visually cluttered backgrounds. In surveillance, the mechanism can distinguish subtle spectral differences between objects and their backgrounds, providing more robust tracking in environments where conventional RGB data might fail.

The channel attention based on adaptive soft threshold proposed in this article significantly improves the discriminative power of network-extracted features. Therefore, it shows stronger robustness in long-term (tracking sequence length exceeds 600 frames) tracking scenarios such as in Figure 9b. Excellent performance, achieving stable target tracking.

As shown in Figure 9d, the adaptive approach in HSATrack tailors the attention mechanism to the specific spectral signatures of the objects being tracked in real-world challenges. This is crucial for complex scenarios such as under varying weather conditions, changes in illumination, and occlusions. By doing so, HSATrack demonstrates the potential to greatly enhance the reliability and accuracy of hyperspectral object tracking systems in real-world operations. In the application of remote sensing image processing, agricultural detection, and military operations, complex application environments such as illumination various and occlusions are often encountered. By using HSATrack, the processing performance in these complex environments can be further enhanced, demonstrating the practical value of the proposed method.

### 4.4. Failure Cases

**Figure 10:**
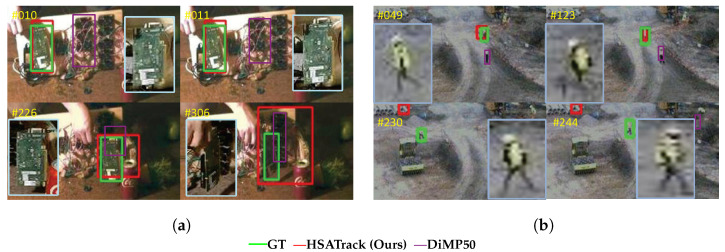
Failure cases of HSATrack in different scenes. Target (circuit boards) in (**a**) experiencing drastic changes of appearance. Target (worker) in (**b**) in scenes with low image quality and blurry background. Different colors represent different algorithm tracking results.

Figure 10a illustrates instances where HSATrack’s tracking performance falters, particularly when the subject undergoes heavy occlusion or marked alterations in its visual characteristics. Such difficulties stem from the tracking system’s difficulty in swiftly recognizing and adapting to the subject’s sudden visual shifts during extended periods of observation. This can result in an inability to refresh the template buffer effectively, thus impairing the tracker’s precision. To address these shortcomings, we plan to integrate modules that are sensitive to occlusions and capable of maintaining robustness across varying scales in our future research endeavors.

As is shown in Figure 10b, in scenes with low image quality and a blurry background, our tracking algorithm will also fail. This is due to the inability to extract effective target features due to poor image quality, and the method in this paper does not solve this challenge, so tracking fails. In response to this problem, follow-up work can consider drawing on ideas from the super-resolution field and using generation methods to improve image quality.

### 4.5. Ablation Study

To substantiate the performance of the core elements within our proposed method, we performed an ablation analysis using the WHISPER2020 dataset. When training our HSATrack, we only used false-color-based images from the WHISPER2020 dataset. Five HSATrack variant structures were obtained: HSATrack with False-Color, HSATrack with HyperSpectral, HSATrack with False-Color-pretrain, HSATrack with No-SAM, and HSATrack with No-Hierarchy structure. A comparison of the results of these trackers id shown in Table 3. We found that the hierarchical structure had the greatest impact on the tracking results, followed by SAM and training data. This is because the hierarchical structure is critical to the integration of spatial information throughout the network, so, without this structure, the performance of the tracker will drop rapidly. Without the SAM module, multispectral images cannot be integrated with multispectral information, so the performance is reduced a lot, which is followed by the impact of various different data sources. The superior performance of our method validates the effectiveness of the hierarchical structure and SAM in hyperspectral video object tracking.

In order to explore the impact of the size of the training dataset on the performance of the algorithm, we randomly selected 35, 30, and 25 groups of training video sequences for testing, constructed HSATrack_35_, HSATrack_30_, HSATrack_25_, and compared them with our method, HSATrack. A comparison of the results is shown in the Table 4. It can be seen that the size of the dataset significantly affects the performance of the tracking model. This is because the tracking model based on deep learning depends on the scale of the training data. The larger the scale, the richer the prior information of the target, which is more conducive to the training of the tracking model.

**Figure 11:**
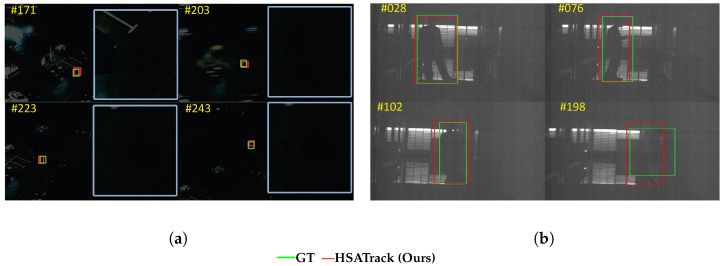
Visualized results of overfitting ablation experiment. Video sequence (**a**) is a cyclist captured by a drone. Video sequence (**b**) is a boy captured by a handheld surveillance camera.

In order to verify whether the method proposed in this article overfits specific datasets, we used drones and cameras from video surveillance perspectives to conduct real-life shooting, and we copied 16 copies of the simulated hyperspectral data from the image channel of the visible RGB image. We performed visual analysis of the images, as shown in Figure 11. Judging from the visualized results, the method proposed in this article achieves stable target tracking, which is enough to prove the robustness of the method proposed in this article and that it does not overfit special datasets.

## 5. Conclusions

In this paper, we propose a hierarchical construction based on spectral attention for hyperspectral object tracking, called HSATrack. HSATrack introduces a hierarchical spectral attention network for hyperspectral object tracking. First, we employ a novel spectral band attention mechanism with an adaptive soft threshold to examine correlations across spectral bands, which integrates the information available in various spectral bands and eliminates redundant information. Second, we integrate spectral attention into a hierarchical tracking network to improve the integration of spectral and spatial information. Our method outperforms existing hyperspectral trackers by taking advantage of the spatial and spectral information of the targets. The processing speed of our method is 40 frames per second, and extensive experiments on WHISPER2020 demonstrate the efficiency and promising performance of our proposed method.

## Figures and Tables

**Table 1:** The AUC scores, DP scores, and FPS of the hyperspectral object trackers. At the same time, we also compared the processing speed of the algorithm on different hardware devices. We selected eight trackers for comparison, and the top two trackers are marked in red and blue.

	CNHT	[12]	MHT	BAE-Net	TransT	STARK	ViPT	SiamHYPER_*BAN*_	DiMP50_*our*_	Our
AUC	0.177	0.421	0.587	0.606	0.476	0.585	0.617	0.684	0.576	0.660
DP	0.334	0.715	0.880	0.877	0.739	0.884	0.906	0.939	0.849	0.918
FPS	0.8	210	0.5	0.5	31	24	17	19	46	34
Hardware	I7	3090	I7	TITAN X	3090	3090	3090	TITAN X	3090	3090

**Table 2:** Attribute comparison of AUC on WHISPER2020 includes various challenging factors such as BC, DEF, FM, IV, IPR, LR, MB, OCC, OPR, OV, SC, and SV. The comparison involves trackers: our method, our baseline DiMP50, CNHT, MHT, and SiamHYPER_*BAN*_. The best two results are shown in red and blue fonts.

Attribute	CNHT	[12]	MHT	TransT	STARK	ViPT	SiamHYPER_*BAN*_	DiMP50_*our*_	Ours
SV	0.214	0.501	0.634	0.622	0.577	0.591	0.596	0.540	0.653
SC	0.38	0.498	0.646	0.582	0.616	0.603	0.767	0.66	0.66
OV	0.149	0.442	0.62	0.581	0.544	0.611	0.596	0.585	0.585
OPR	0.264	0.569	0.724	0.664	0.619	0.677	0.752	0.719	0.719
OCC	0.123	0.474	0.564	0.522	0.60	0.601	0.635	0.588	0.625
MB	0.102	0.381	0.56	0.589	0.688	0.693	0.75	0.664	0.707
LR	0.029	0.299	0.477	0.539	0.556	0.578	0.664	0.553	0.58
IPR	0.278	0.510	0.67	0.599	0.655	0.673	0.72	0.631	0.678
IV	0.087	0.312	0.473	0.582	0.571	0.591	0.592	0.546	0.651
FM	0.189	0.451	0.541	0.592	0.622	0.633	0.71	0.643	0.643
DEF	0.294	0.577	0.664	0.591	0.692	0.700	0.72	0.686	0.778
BC	0.183	0.444	0.594	0.572	0.676	0.683	0.702	0.561	0.685
FPS	0.8	210	0.5	31	24	17	19	46	34

**Table 3:** Ablation analysis (AUC/DP) of HSATrack variants. In order to explore the impact of different modules on the algorithm, we set up five variant algorithm ablation experiments. The top two trackers are marked in red and blue.

Method	WHISPER_*AUC*_	WHISPER_*DP*_
HSATrack_*False*−*Color*_	0.302	0.621
HSATrack_*HyperSpectral*_	0.221	0.579
HSATrack_*False*−*Color*−*pretrain*_	0.413	0.625
HSATrack_*No*−*SAM*_	0.557	0.820
HSATrack_*No*−*Heri*_	0.501	0.865
Our	0.66	0.918

**Table 4:** Ablation analysis (AUC/DP) of HSATrack variants with different training data. The top two trackers are marked in red and blue.

Method	WHISPER_*AUC*_	WHISPER_*DP*_
HSATrack_25_	0.012	0.103
HSATrack_30_	0.244	0.418
HSATrack_35_	0.475	0.785
Our	0.66	0.918

## Data Availability

WHISPERS is a conference on hyperspectral target tracking that has been held for three years since 2020. The public dataset released by the conference is called WHISPERS, also known as HOT. However, the dataset has not been updated during these three years, so the industry refers to the hyperspectral dataset as WHISPER2020 or HOT2020. The WHISPER2020 competition dataset used in this work is listed here: https://www.hsitracking.com/hot2020/, accessed on 22 September 2024. The Baidu cloud link is listed here: https://pan.baidu.com/s/1FY2L6L9SDKw-V-bUkuosSA/, accessed on 22 September 2024.

## References

[1] Zhang H., Li Y., Liu H., Yuan D., Yang Y. (2023). Learning Response-Consistent and Background-Suppressed Correlation Filters for Real-Time UAV Tracking. Sensors.

[2] Gora O., Akkan T. (2023). Development of a Novel Spherical Light-Based Positioning Sensor in Solar Tracking. Sensors.

[3] Corera Í., Piñeiro E., Navallas-Irujo J., Sagues M., Loayssa A. (2023). Long-Range Traffic Monitoring Based on Pulse-Compression Distributed Acoustic Sensing and Advanced Vehicle Tracking and Classification Algorithm. Sensors.

[4] Zheng X., He T. (2023). Reduced-Parameter YOLO-like Object Detector Oriented to Resource-Constrained Platform. Sensors.

[5] Ogunrinde I.O., Bernadin S. (2024). Improved DeepSORT-Based Object Tracking in Foggy Weather for AVs Using Sematic Labels and Fused Appearance Feature Network. Sensors.

[6] Yi A., Anantrasirichai N. (2024). A Comprehensive Study of Object Tracking in Low-Light Environments. Sensors.

[7] Zhu Y., An H., Wang H., Xu R., Sun Z., Lu K. (2024). DOT-SLAM: A Stereo Visual Simultaneous Localization and Mapping (SLAM) System with Dynamic Object Tracking Based on Graph Optimization. Sensors.

[8] Li B., Wu W., Wang Q., Zhang F., Xing J., Yan J. Siamrpn++: Evolution of siamese visual tracking with very deep networks. Proceedings of the IEEE/CVF Conference on Computer Vision and Pattern Recognition.

[9] Zhang K., Zhang L., Liu Q., Zhang D., Yang M. Fast Visual Tracking via Dense Spatio-temporal Context Learning. Proceedings of the Computer Vision—ECCV 2014—13th European Conference.

[10] Bolme D.S., Beveridge J.R., Draper B.A., Lui Y.M. Visual object tracking using adaptive correlation filters. Proceedings of the Twenty-Third IEEE Conference on Computer Vision and Pattern Recognition, CVPR.

[11] Chen X., Yan B., Zhu J., Wang D., Yang X., Lu H. Transformer tracking. Proceedings of the IEEE/CVF Conference on Computer Vision and Pattern Recognition.

[12] Kou R., Wang C., Yu Y., Peng Z., Huang F., Fu Q. (2023). Infrared Small Target Tracking Algorithm via Segmentation Network and Multistrategy Fusion. IEEE Trans. Geosci. Remote Sens..

[13] Liu Z., Wang X., Zhong Y., Shu M., Sun C. (2022). SiamHYPER: Learning a hyperspectral object tracker from an RGB-based tracker. IEEE Trans. Image Process..

[14] Manolakis D., Shaw G.A. (2001). Hyperspectral Imaging for Remote Sensing. IEEE Signal Process. Mag..

[15] Lv H., Zhang H., Wang M., Xu J., Li X., Liu C. (2024). Hyperspectral Imaging Based Nonwoven Fabric Defect Detection Method Using LL-YOLOv5. IEEE Access.

[16] Ma R., Ma T., Guo D., He S. (2024). Novel View Synthesis and Dataset Augmentation for Hyperspectral Data Using NeRF. IEEE Access.

[17] Mirza H.I., Ahmed S.B., Solis-Oba R., Malik M.I. (2024). Endmember Analysis of Overlapping Handwritten Text in Hyperspectral Document Images. IEEE Access.

[18] Bioucas-Dias J.M., Plaza A., Camps-Valls G., Scheunders P., Nasrabadi N., Chanussot J. (2013). Hyperspectral Remote Sensing Data Analysis and Future Challenges. IEEE Geosci. Remote Sens. Mag..

[19] Chang C.I. (2006). Anomaly Detection and Classification for Hyperspectral Imagery. IEEE Trans. Geosci. Remote Sens..

[20] Aliouat A., Kouadria N., Maimour M., Harize S. (2024). EVBS-CAT: Enhanced video background subtraction with a controlled adaptive threshold for constrained wireless video surveillance. J. Real Time Image Process..

[21] Aliouat A., Kouadria N., Maimour M., Harize S., Doghmane N. (2023). Region-of-interest based video coding strategy for rate/energy-constrained smart surveillance systems using WMSNs. Ad Hoc Netw..

[22] Aliouat A., Kouadria N., Harize S., Maimour M. (2023). An Efficient Low Complexity Region-of-Interest Detection for Video Coding in Wireless Visual Surveillance. IEEE Access.

[23] Stein D.W., Beaven S.G., Hoff L.E., Winter E.M., Schaum A.P., Stocker A.D. (2002). Real-Time Hyperspectral Image Processing: An Overview. IEEE Trans. Geosci. Remote Sens..

[24] Confalonieri R., Htun P.P., Sun B., Tillo T. (2024). An End-to-End Framework for the Classification of Hyperspectral Images in the Wood Domain. IEEE Access.

[25] Chen Y., Lin Z., Zhao X., Wang G., Gu Y. (2014). Deep Learning for Hyperspectral Image Classification: An Overview. IEEE Trans. Geosci. Remote Sens..

[26] Zhang L., Du B., Zhang L. (2016). Deep Learning for Remote Sensing Data: A Technical Tutorial on the State of the Art. IEEE Geosci. Remote Sens. Mag..

[27] Liu Z., Wang X., Shu M., Li G., Sun C., Liu Z., Zhong Y. An anchor-free Siamese target tracking network for hyperspectral video. Proceedings of the 2021 11th Workshop on Hyperspectral Imaging and Signal Processing: Evolution in Remote Sensing (WHISPERS).

[28] Song W., Jiao L., Liu F., Liu X., Li L., Yang S., Hou B., Zhang W. (2022). A joint siamese attention-aware network for vehicle object tracking in satellite videos. IEEE Trans. Geosci. Remote Sens..

[29] Li C., Zhang J., Wang Y. (2023). SMTN: Multidimensional Fusion and Time Domain Coding for Object Tracking in Satellite Videos. IEEE Geosci. Remote Sens. Lett..

[30] Sun H., Ma P., Li Z., Ye Z., Ma Y. (2024). Hyperspectral low altitude UAV target tracking algorithm based on deep learning and improved KCF. Front. Phys..

[31] Zhao D., Cao J., Zhu X., Zhang Z., Arun P.V., Guo Y., Qian K., Zhang L., Zhou H., Hu J. (2022). Hyperspectral video target tracking based on deep edge convolution feature and improved context filter. Remote Sens..

[32] Qian K., Wang S., Zhang S., Shen J. (2023). SiamPKHT: Hyperspectral Siamese Tracking Based on Pyramid Shuffle Attention and Knowledge Distillation. Sensors.

[33] Ding Y., Liu Y., Soatto S., Jin Y. (2019). A Survey on Deep Learning Techniques for Stereo-Based Depth Estimation. IEEE Trans. Pattern Anal. Mach. Intell..

[34] Bhat G., Danelljan M., Gool L.V., Timofte R. Learning discriminative model prediction for tracking. Proceedings of the IEEE/CVF International Conference on Computer Vision.

[35] Rodarmel C., Shan J. (2002). Principal component analysis for hyperspectral image classification. Surv. Land Inf. Sci..

[36] Van Nguyen H., Banerjee A., Chellappa R. Tracking via object reflectance using a hyperspectral video camera. Proceedings of the 2010 IEEE Computer Society Conference on Computer Vision and Pattern Recognition-Workshops.

[37] Xiong F., Zhou J., Qian Y. (2020). Material based object tracking in hyperspectral videos. IEEE Trans. Image Process..

[38] Qian K., Zhou J., Xiong F., Zhou H., Du J. Object Tracking in Hyperspectral Videos with Convolutional Features and Kernelized Correlation Filter. Proceedings of the Smart Multimedia—First International Conference, ICSM 2018.

[39] Tang Y., Liu Y., Huang H. (2022). Target-aware and spatial-spectral discriminant feature joint correlation filters for hyperspectral video object tracking. Comput. Vis. Image Underst..

[40] Uzkent B., Rangnekar A., Hoffman M.J. (2018). Tracking in aerial hyperspectral videos using deep kernelized correlation filters. IEEE Trans. Geosci. Remote Sens..

[41] Li Y., Zhang H., Shen Q. (2017). Spectral–spatial classification of hyperspectral imagery with 3D convolutional neural network. Remote Sens..

[42] Li Z., Xiong F., Zhou J., Wang J., Lu J., Qian Y. BAE-Net: A band attention aware ensemble network for hyperspectral object tracking. Proceedings of the 2020 IEEE International Conference on Image Processing (ICIP).

[43] Song Y., Ma C., Wu X., Gong L., Bao L., Zuo W., Shen C., Lau R.W., Yang M.H. Vital: Visual tracking via adversarial learning. Proceedings of the IEEE Conference on Computer Vision and Pattern Recognition.

[44] Li Z., Ye X., Xiong F., Lu J., Zhou J., Qian Y. Spectral-Spatial-Temporal attention network for hyperspectral tracking. Proceedings of the 2021 11th Workshop on Hyperspectral Imaging and Signal Processing: Evolution in Remote Sensing (WHISPERS).

[45] Wang H., Li W., Xia X., Du Q., Tian J., Shen Q. (2024). Transformer-Based Band Regrouping With Feature Refinement for Hyperspectral Object Tracking. IEEE Trans. Geosci. Remote Sens..

[46] Vaswani A., Shazeer N., Parmar N., Uszkoreit J., Jones L., Gomez A.N., Kaiser L., Polosukhin I. Attention is All you Need. Proceedings of the Advances in Neural Information Processing Systems 30: Annual Conference on Neural Information Processing Systems 2017.

[47] Li Y., Luo Y., Zhang L., Wang Z., Du B. (2024). MambaHSI: Spatial-Spectral Mamba for Hyperspectral Image Classification. IEEE Trans. Geosci. Remote Sens..

[48] Gu A., Dao T. (2023). Mamba: Linear-Time Sequence Modeling with Selective State Spaces. arXiv.

[49] Zhang X., Sun Y., Jiang K., Li C., Jiao L., Zhou H. (2018). Spatial sequential recurrent neural network for hyperspectral image classification. IEEE J. Sel. Top. Appl. Earth Obs. Remote Sens..

[50] Haut J.M., Paoletti M.E., Plaza J., Plaza A., Li J. (2019). Visual attention-driven hyperspectral image classification. IEEE Trans. Geosci. Remote Sens..

[51] Stone H.S., Orchard M.T., Chang E., Martucci S.A. (2001). A fast direct Fourier-based algorithm for subpixel registration of images. IEEE Trans. Geosci. Remote Sens..

[52] Ren J., Vlachos T., Zhang Y., Zheng J., Jiang J. (2014). Gradient-based subspace phase correlation for fast and effective image alignment. J. Vis. Commun. Image Represent..

[53] Gao W., Tian X., Zhang Y., Jia N., Yang T., Jiao L. Dual Siamese Channel Attention Networks for Visual Object Tracking. Proceedings of the Intelligence Science IV—5th IFIP TC 12 International Conference, ICIS 2022.

[54] Zhang Y., Yu Y., Huang K., Wang Y. (2023). Channel Attentional Correlation Filters Learning With Second-Order Difference for UAV Tracking. IEEE Geosci. Remote Sens. Lett..

[55] He K., Zhang X., Ren S., Sun J. Deep residual learning for image recognition. Proceedings of the IEEE Conference on Computer Vision and Pattern Recognition.

[56] Bayraktar E., Wang Y., DelBue A. (2022). Fast re-OBJ: Real-time object re-identification in rigid scenes. Mach. Vis. Appl..

[57] Bayraktar E. (2023). Improved Object Re-Identification via More Efficient Embeddings. Turk. J. Electr. Eng. Comput. Sci..

[58] Kingma D.P., Ba J. Adam: A method for stochastic optimization. Proceedings of the 3rd International Conference for Learning Representations.

[59] Yan B., Peng H., Fu J., Wang D., Lu H. Learning spatio-temporal transformer for visual tracking. Proceedings of the IEEE/CVF International Conference on Computer Vision.

[60] Zhu J., Lai S., Chen X., Wang D., Lu H. Visual prompt multi-modal tracking. Proceedings of the IEEE/CVF Conference on Computer Vision and Pattern Recognition.

[61] Chen Z., Zhong B., Li G., Zhang S., Ji R. Siamese box adaptive network for visual tracking. Proceedings of the IEEE/CVF Conference on Computer Vision and Pattern Recognition.

